# Supplementary material for: Cortical oscillations reflect opponent ensemble dynamics through coordinated multifrequency activity
Source: bioRxiv. 2026 Feb 23:2026.02.20.707132. Preprint. [Version 1] doi: 10.64898/2026.02.20.707132 (PMC12970329; doi:10.64898/2026.02.20.707132)
Supplement: Supplementary file 1 [file NIHPP2026.02.20.707132v1-supplement-1.pdf]

bioRxiv preprint doi: https://doi.org/10.64898/2026.02.20.707132; this version posted February 23, 2026. The copyright holder for this preprint (which was not certified by peer review) is the author/funder. This article is a US Government work. It is not subject to copyright under 17 USC 105 and is also made available for use under a CC0 license.

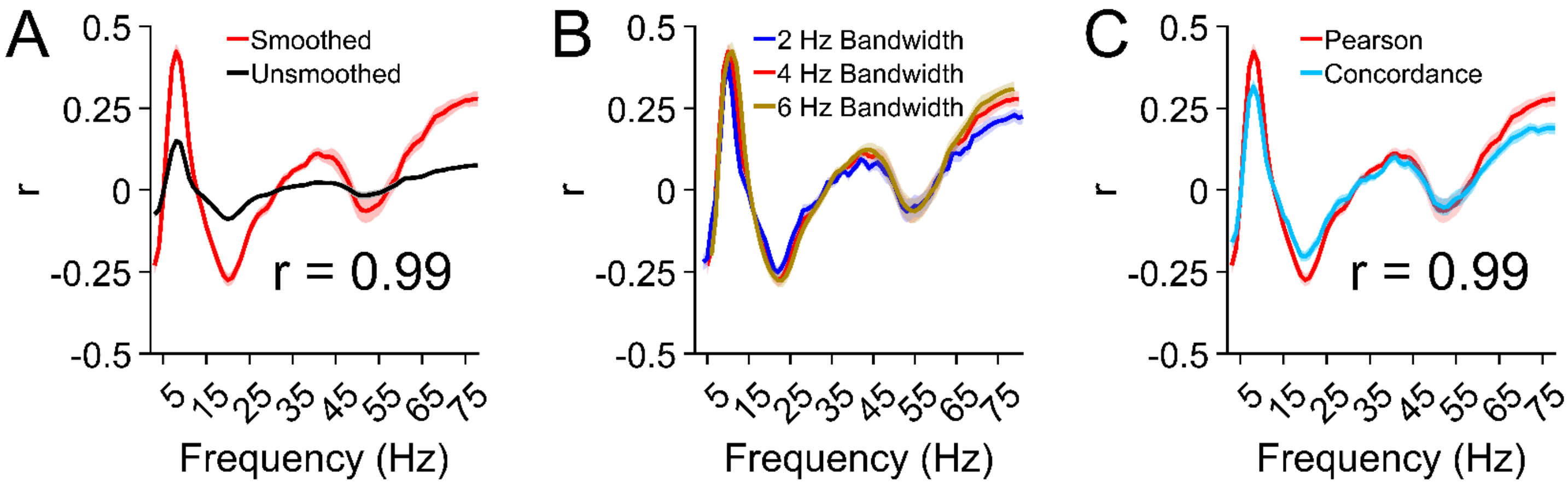

Supplementary Figure 1. Frequency–calcium relationships are robust to amplitude smoothing and bandwidth definition. (A) Comparison of smoothed and unsmoothed band-limited LFP amplitude envelopes used to compute frequency-calcium relationships. (B) Frequency-specific correlations computed using different bandwidth definitions (2 Hz, blue; 4 Hz, red; 6 Hz, gold), demonstrating preservation of spectral structure across bandwidth choices. (C) Frequency-calcium relationships quantified using Pearson correlation (red) and mean concordance (light blue, see Methods), showing consistent spectral organization across metrics.

bioRxiv preprint doi: https://doi.org/10.64898/2026.02.20.707132; this version posted February 23, 2026. The copyright holder for this preprint (which was not certified by peer review) is the author/funder. This article is a US Government work. It is not subject to copyright under 17 USC 105 and is also made available for use under a CC0 license.

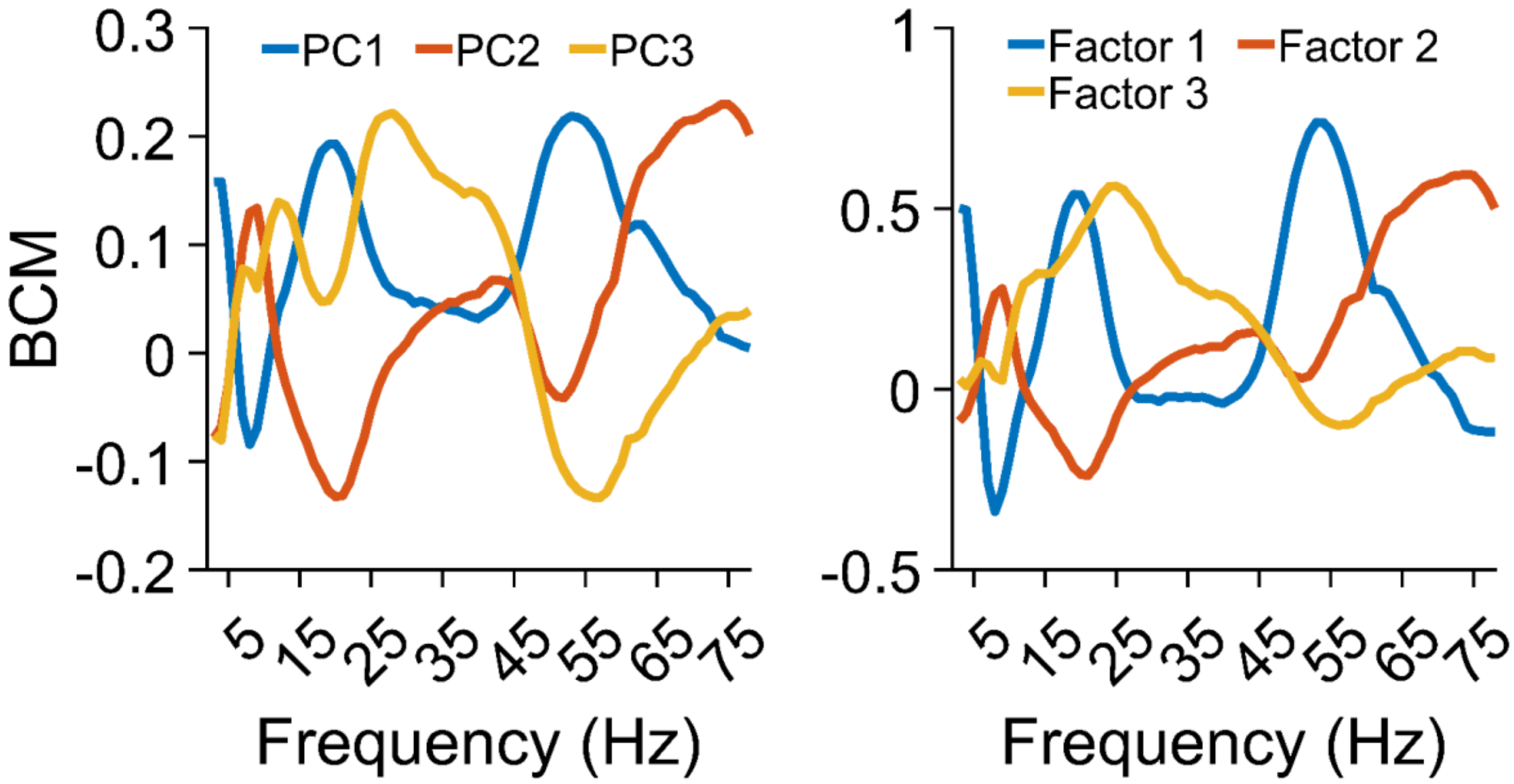

Supplementary Figure 2. Opponent spectral motif structure is preserved across factorization methods. Recurring multiband amplitude configurations identified directly from band-limited LFP concordance with calcium activity (see Methods) using PCA and factor analysis.

bioRxiv preprint doi: https://doi.org/10.64898/2026.02.20.707132; this version posted February 23, 2026. The copyright holder for this preprint (which was not certified by peer review) is the author/funder. This article is a US Government work. It is not subject to copyright under 17 USC 105 and is also made available for use under a CC0 license.

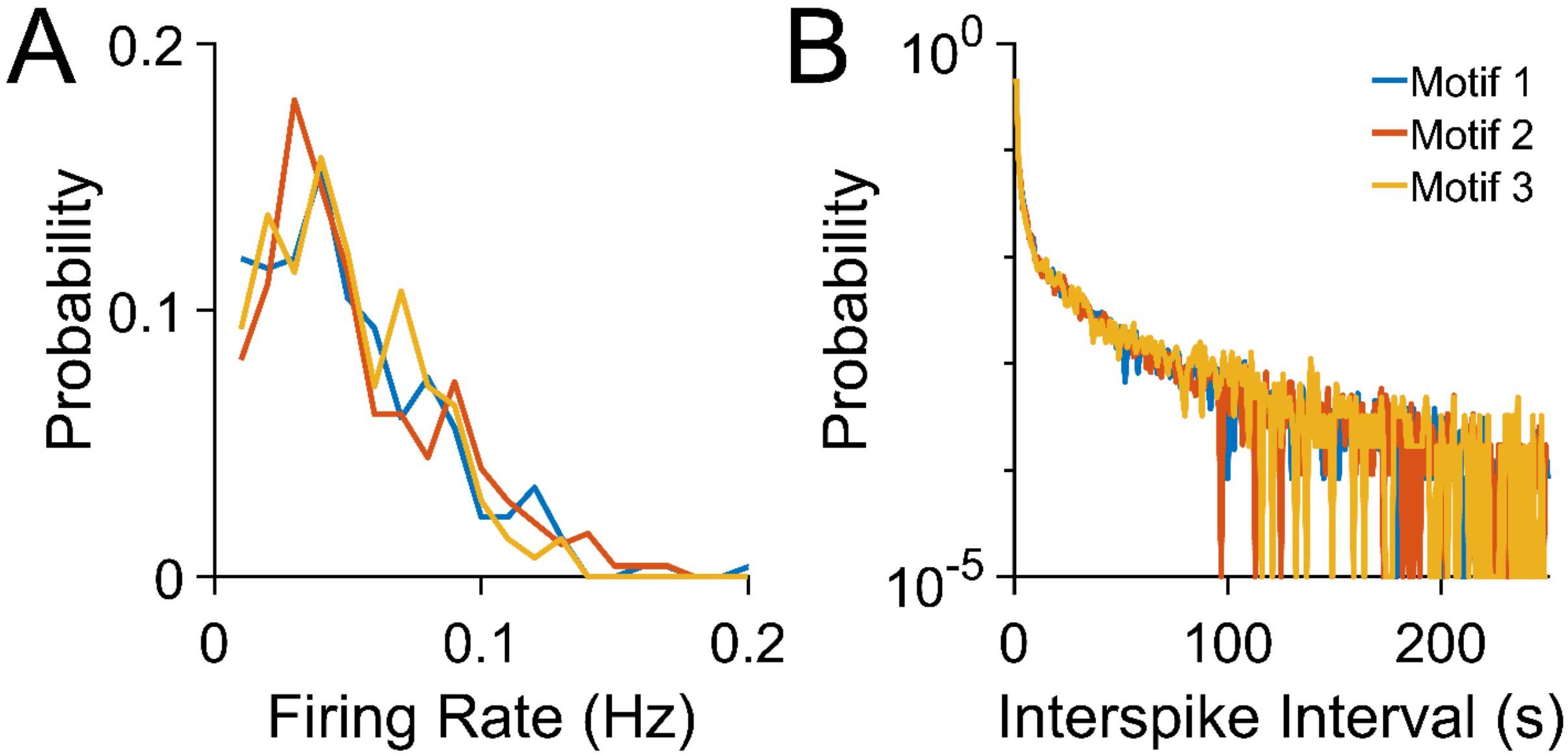

Supplementary Figure 3. Motif-defined ensembles are not distinguished by gross firing statistics. (A) Distribution of mean firing rates for neurons assigned to each motif. No consistent differences were observed across sessions. (B) Interspike interval (ISI) distributions for neurons grouped by opponent motif assignment. ISI structure did not systematically differ across motifs.

bioRxiv preprint doi: https://doi.org/10.64898/2026.02.20.707132; this version posted February 23, 2026. The copyright holder for this preprint (which was not certified by peer review) is the author/funder. This article is a US Government work. It is not subject to copyright under 17 USC 105 and is also made available for use under a CC0 license.

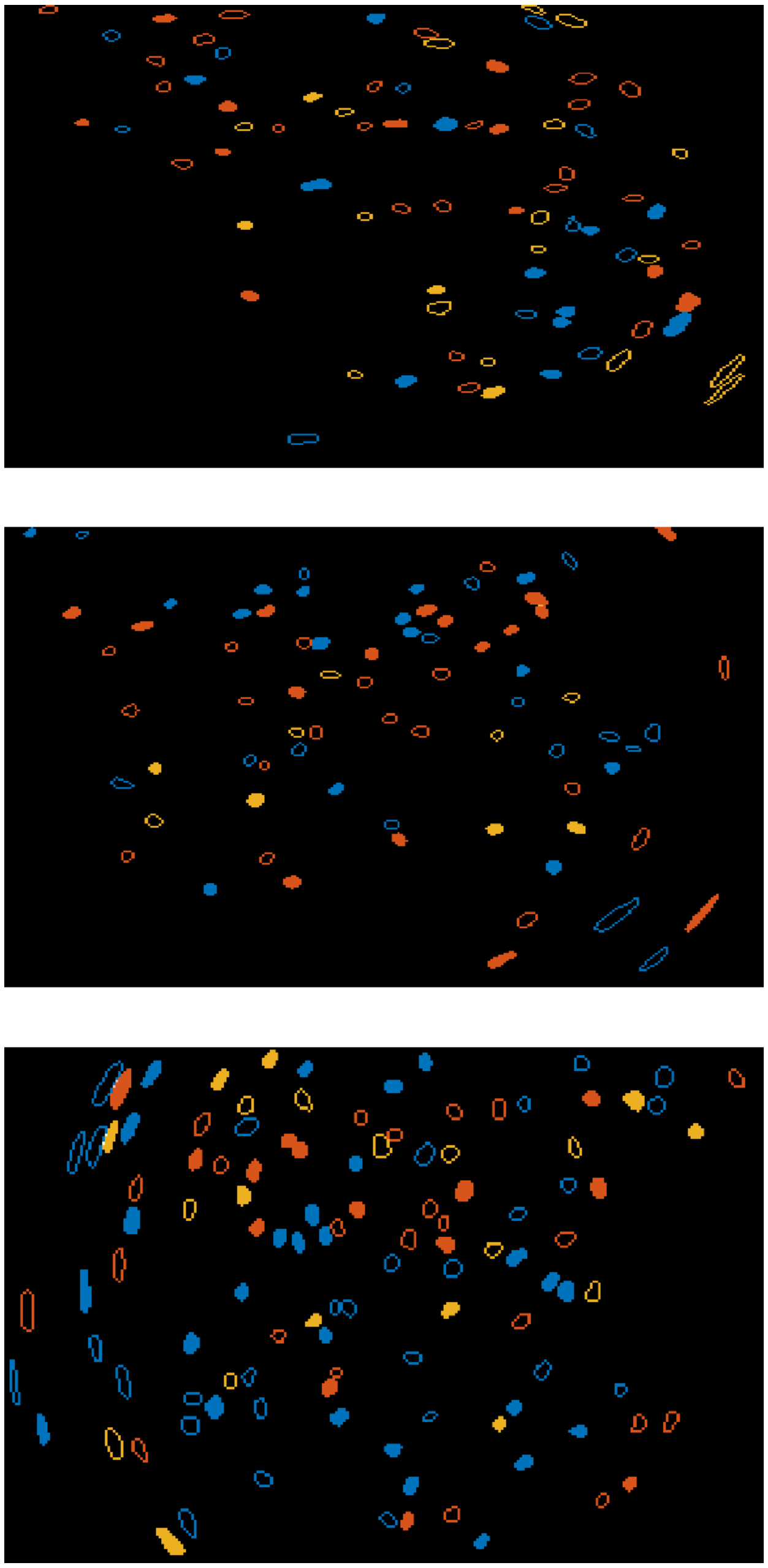

Supplementary Figure 4. Opponent neuronal ensembles are spatially intermingled within mPFC. Spatial distribution of neurons assigned to Motif 1 (blue), Motif 2 (orange), and Motif 3 (yellow) across three sessions from three rats. Open and closed markers denote opposing A and B components within each motif. Neurons belonging to opponent components were interspersed throughout the imaging field rather than anatomically clustered. Nearest-neighbor permutation analyses revealed minimal deviation from chance organization, with small effect sizes and few significant motif-pair comparisons relative to shuffled controls (97.6% of permutations $p > 0.05$), indicating that motif-defined ensembles reflect functional rather than anatomical segregation.

bioRxiv preprint doi: https://doi.org/10.64898/2026.02.20.707132; this version posted February 23, 2026. The copyright holder for this preprint (which was not certified by peer review) is the author/funder. This article is a US Government work. It is not subject to copyright under 17 USC 105 and is also made available for use under a CC0 license.

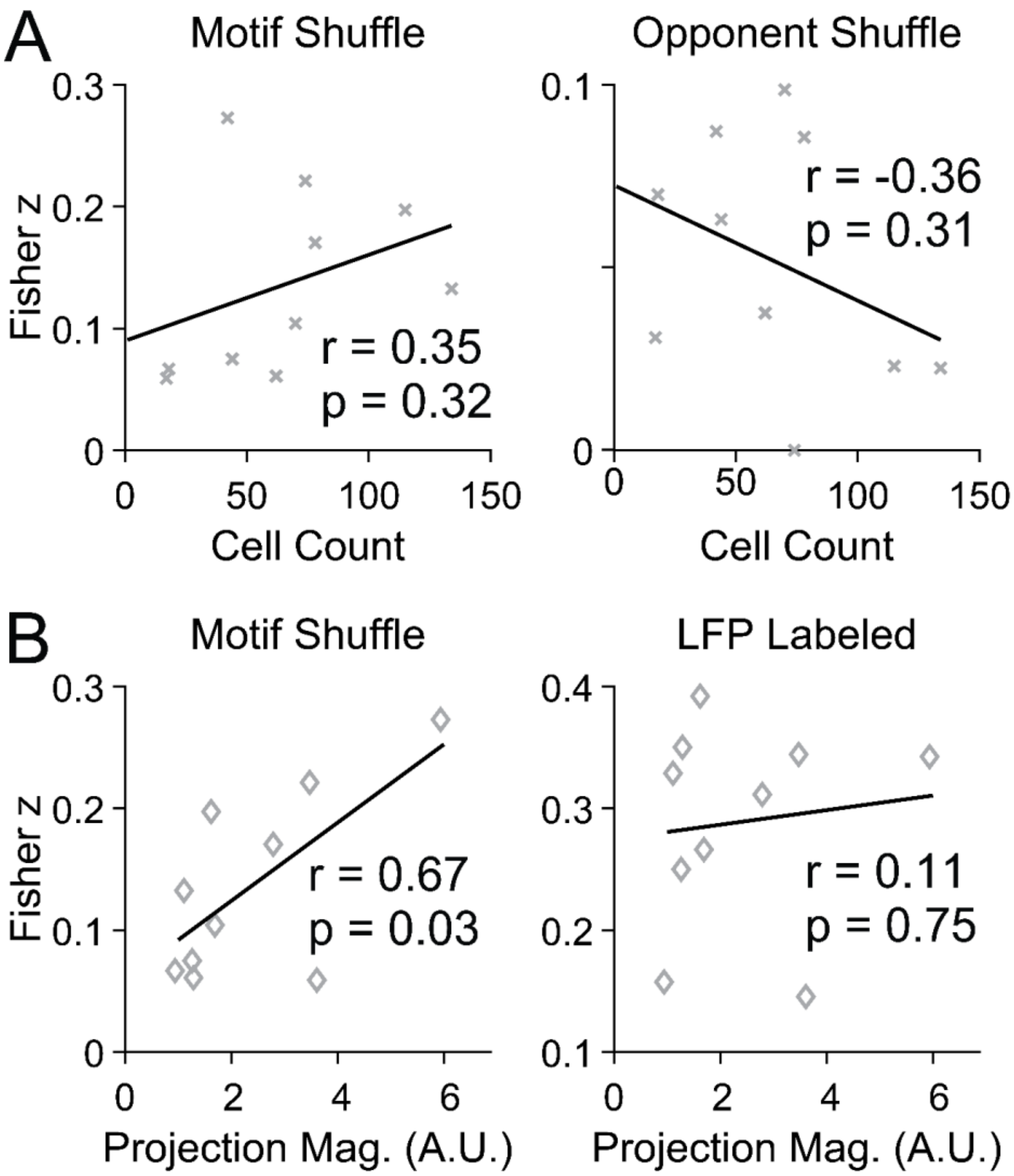

Supplementary Figure 5. Control reconstructions fail to scale with cell count and remain dependent on opponent motif dominance. (A) Relationship between reconstruction accuracy and number of recorded neurons per session for control models. Motif-shuffled (left) and opponent-shuffled (right) reconstructions did not scale with cell count (motif-shuffled: $r = 0.35$, $p = 0.32$; opponent-shuffled: $r = −0.36$, $p = 0.31$). (B) Dependence of reconstruction accuracy on relative dominance of A versus B opponent motifs. Motif-shuffled reconstructions showed a dependence of A-motif dominance ($r = 0.67$, $p = 0.03$, left). In contrast, reconstructions preserving motif and opponent identity were invariant to opponent balance ($r = 0.11$, $p = 0.75$, right), indicating that accurate reconstruction depends on opponent structure rather than global shifts in ensemble activity.

bioRxiv preprint doi: https://doi.org/10.64898/2026.02.20.707132; this version posted February 23, 2026. The copyright holder for this preprint (which was not certified by peer review) is the author/funder. This article is a US Government work. It is not subject to copyright under 17 USC 105 and is also made available for use under a CC0 license.

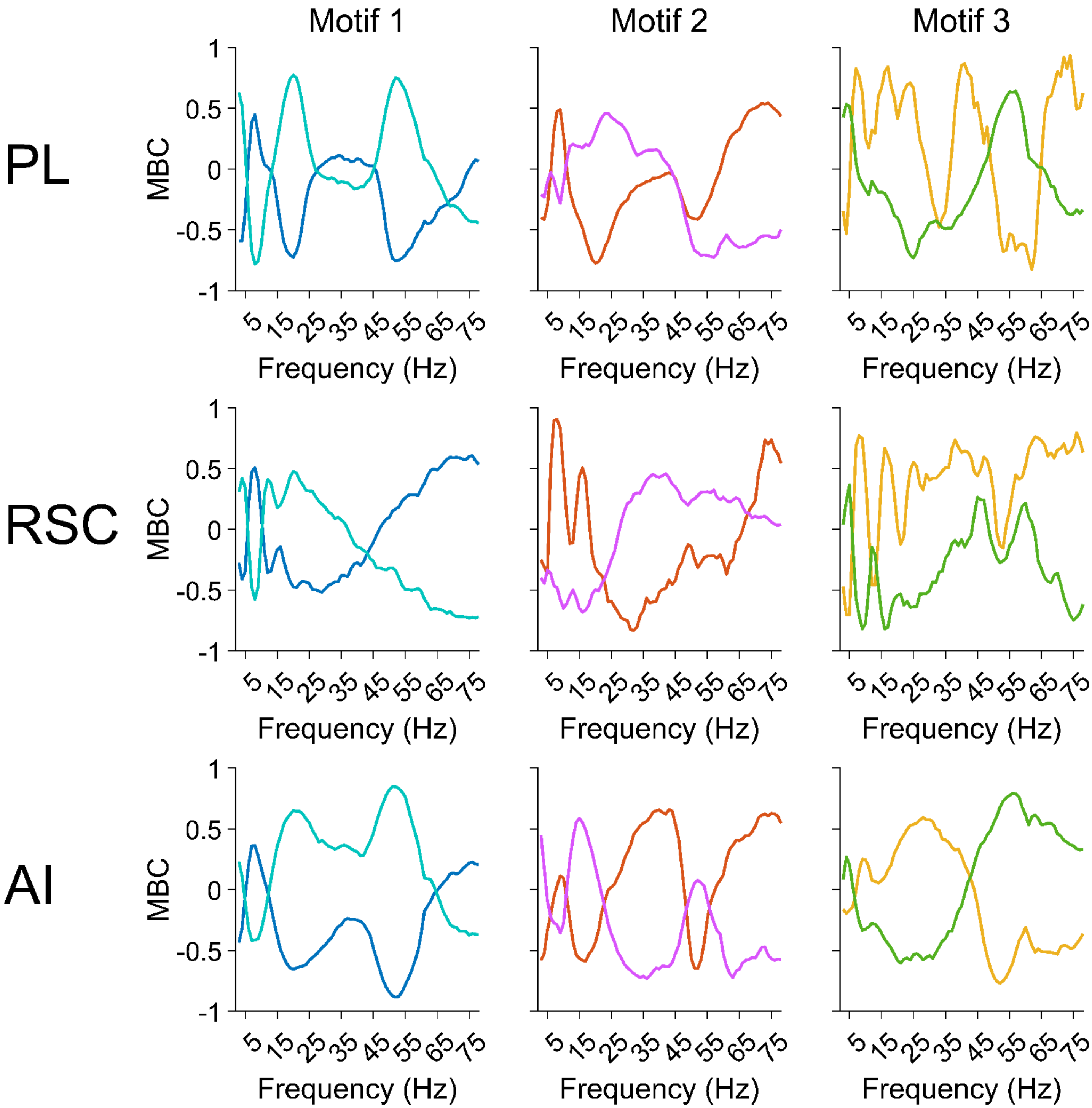

Supplementary Figure 6. Opponent spectral motifs are recoverable from multiband LFP dynamics and vary across cortical regions. (Top) Opponent spectral motifs identified using LFP data alone by clustering multiband amplitude co-fluctuations without reference to calcium activity. (Middle) LFP-only motif extraction in RSC. (Bottom) LFP-only motif extraction in AI.

bioRxiv preprint doi: https://doi.org/10.64898/2026.02.20.707132; this version posted February 23, 2026. The copyright holder for this preprint (which was not certified by peer review) is the author/funder. This article is a US Government work. It is not subject to copyright under 17 USC 105 and is also made available for use under a CC0 license.

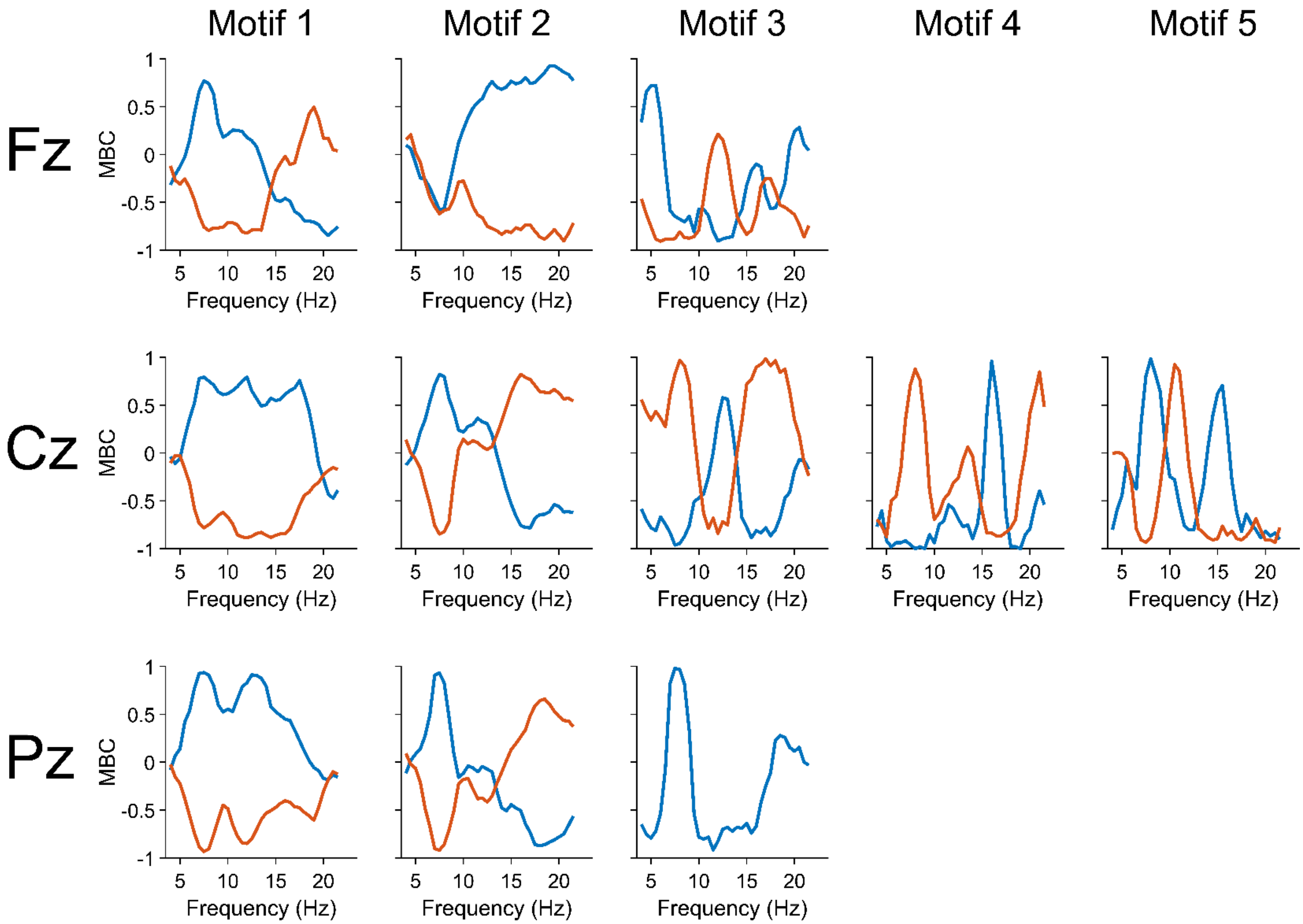

Supplementary Figure 7. Opponent multiband spectral motifs generalize to noninvasive human EEG. Spectral motifs were extracted from midline EEG electrodes (Fz, Cz, and Pz) across 10 human subjects using the same multiband amplitude co-fluctuation procedure applied to rodent LFP recordings. Motif structure was reproducible across subjects and electrode sites, and recapitulated opponent dynamics observed in rats.
